# The Integration of Patient-Reported Quality of Life and Systemic Biomarkers in Patients with Immune Dysregulation

**DOI:** 10.21203/rs.3.rs-3270389/v1

**Published:** 2023-08-23

**Authors:** Brenna LaBere, Anne Chu, Craig D. Platt, Janet Chou

**Affiliations:** Phoenix Children’s Hospital; Boston Children’s Hospital; Boston Children’s Hospital; Boston Children’s Hospital

**Keywords:** Immune dysregulation, Quality of life, T follicular helper cell

## Abstract

**Background:**

Patient-reported quality of life measurements are an important method for improving the treatment of patients with a variety of diseases. These tools have been minimally investigated in patients with inborn errors of immunity (IEI). Patients with IEI may have immune dysregulation and autoimmune-mediated multi-system organ involvement, making treatment optimization vitally important. Routine laboratory and radiologic testing are typically used for treatment monitoring; however, these modalities have the potential to miss early organ damage. T follicular helper cells are T cells that contribute to antibody production and are known to be expanded in patients with active autoimmunity. We hypothesized that a combination of patient-reported quality of life measurements, in addition to T follicular helper cell percentages, would help us to better understand the level of disease activity in patients with IEI and autoimmunity.

**Methods:**

Patients with immune dysregulation were consented to provide a blood sample and to complete a questionnaire. The Centers for Disease Control HRQOL-14 tool was utilized for the questionnaire portion, and T follicular helper cell levels were measured from whole blood using surface staining and flow cytometry analysis. Patient disease activity was abstracted from the patient medical record, and this was compared to the questionnaire and whole blood assay results.

**Results:**

A total of 20 patients participated in the study; 8 patients had active disease and the remaining were found to be quiescent. There was no significant difference between the patient-reported general health ratings based on sex, age, disease activity, or category of immune dysregulation (*p* > 0.05). The cTfh percentages were expanded in patients with active disease as compared to those with quiescent (*p* < 0.05). However, there was no significant correlation between cTfh percentage and patient-reported unhealthy days from the questionnaire (R^2^ = 0.113, *p* > 0.05).

**Conclusions:**

Patients with active immune dysregulation were found to have expanded cTfh percentages as compared to those with quiescent disease, however this was not reflected in patient-reported quality of life questionnaires. Better understanding of disease activity and the patient experience is vital to optimize appropriate treatments and outcomes for patients with IEI and immune dysregulation, and more investigation is needed.

## Background

Health-related quality of life (HRQOL) measurements investigate the physical and psychosocial domains of health as determined by a combination of subjective and objective data.^1^ It is known that provider perception of the patient HRQOL is flawed,^2,3^ with discordance demonstrated in patients with inborn errors of immunity (IEI) in particular.^4^ Immune dysregulation, characterized by autoimmunity, autoinflammation, or immune-mediated organ dysfunction, is increasingly recognized as a significant feature of IEI.^5^ Patients with IEI and autoimmune disease can experience significant negative impacts on their physical, mental, and emotional well-being, and this can affect their HRQOL^6–9^. Investigating this aspect of the patient experience may allow for improvement in shared decision-making regarding optimal therapeutic management over time^1^. Few studies have investigated the HRQOL in pediatric patients with IEI^4,6–9^ and others have investigated only organ-specific autoimmune diseases, such as autoimmune hepatitis or immune thrombocytopenia (ITP).^10–12^ However, there are no known studies investigating HRQOL with immune dysregulation in particular. Given the increased recognition for patients with these heterogeneous conditions, this is an important area of research that deserves further investigation.

Recognition of immune dysregulation is challenging due to phenotypic variability and a paucity of biomarkers that quantify disease activity.^13–16^ Circulating T follicular helper cells (cTfh) are a subset of CD4+ T cells essential for an appropriate antibody response.^17^ Patients with autoimmunity have been found to have elevated percentages of cTfh cells, and in some studies these have been representative of disease activity.^13–16,18–22^ However, this marker is not commonly used on a clinical basis due to a lack of widespread clinical validation.

Although comparisons between level of disease control and patient-reported quality of life scores have been evaluated in other diseases, such as rheumatoid arthritis,^23^ few have compared patient-reported quality of life metrics to laboratory biomarkers in prognosticating disease severity in IEI.^6^ Given the unpredictable course of immune dysregulation, better tools are needed for measuring disease progression prior to the development of end-organ damage. We hypothesized that for patients with active immune dysregulation, as compared to those with quiescent disease, that 1) patient-reported quality of life scores would be lower, and 2) cTfh percentages would be elevated. Furthermore, we predicted that the integration of patient-reported quality of life scores with cTfh percentage would provide a better understanding of disease activity in patients with immune dysregulation as compared with either measurement alone.

## Methods

### Study sample:

Patients were recruited at Boston Children’s Hospital. The study was approved by the Institutional Review Board of Boston Children’s Hospital. Study participants were recruited if they had monogenic or polygenic disorders of immune dysregulation, all of which included autoimmunity and many with associated immune deficiency. Of 67 total patients recruited to the study, a total of 20 patients consented to both blood work and completion of the HRQOL survey. All patients had cTfh levels measured during the same week that they completed the HRQOL survey.

Patient race and ethnicity were self-designated in the patient chart. Disease activity was determined from the patient’s medical record by the study’s clinical immunologists prior to measurement of cTfh cells. Active disease was defined by the presence of symptoms, examination findings, laboratory, radiologic, or other diagnostic abnormalities indicative of active disease, as reported by medical providers in the electronic medical record. Quiescent disease was defined by an absence of physical complaints or changes in laboratory, radiologic, or other diagnostic testing in the patients’ medical records, as compared to the patient’s baseline clinical status.

### HRQOL Surveys:

Patients were provided with the Centers for Disease Control (CDC) HRQOL-14 survey (**Supplemental material**), which was either completed on paper or electronically via email after consent was obtained during the patient’s clinic visit. The survey was completed by a parent if the patient was younger than 13 years of age, and by the patient themselves if 13 years and older, with a combination of parental consent and patient assent to participate. The HRQOL-14 survey is composed of four core questions that make up the “Healthy Days Measures” in addition to 10 additional questions that delve into specific quality of life factors that may be affected by a patient’s health.^24^

For the analysis, we focused on the core Healthy Days Core Module questions, which includes the patient’s perception of their general health, total number of unhealthy days over the preceding month, number of physically unhealthy days, and number of mentally unhealthy days. The total number of unhealthy days was determined by adding the number of physically unhealthy days and mentally unhealthy days, with a maximum number of 30 days out of the preceding month, per the CDC HRQOL-14 methods.^24^

### Flow cytometry:

Surface staining for CD4 (BD, Cat# 347413), PD-1 (BioLegend, Cat# 329907), and CXCR5 (Invitrogen, Cat# 12-9185-42) was performed on whole blood by incubating with monoclonal antibodies for 15 minutes, followed by lysis in BD FACS Lysing Solution (diluted 1:10 in MilliQ water). Cells were centrifuged at 1500 rpm for 5 minutes, followed by resuspension in 200 uL phosphate buffer saline. Data were collected with a Fortessa cytometer (BD Biosciences) and analyzed with FlowJo software (TreeStar, Ashland, Ore). PD-1^+^CXCR5^+^ cells were defined as a percentage of the total CD4^+^ T cell population, with a stop gate of 10,000 CD3^+^ events.

### Statistical analyses:

Comparison between groups was carried out with Mann-Whitney test and multi-comparison Kruskal-Wallis tests, as indicated. A simple linear regression and Spearman rank correlation test were performed to assess the relationship between cTfh percentage and number of unhealthy days. Differences were considered significant at a p value of less than 0.05, with adjustment for multiple comparisons as indicated.

## Results

### Demographics:

There were 20 total participants included in the study, with 60% of participants being female (Table 1). Patients were divided by age group, with most patients being over 16 years of age, at 35%. Most patients were white and non-Hispanic, with three patients being Hispanic/Latino (n=2) or Black/African American (n=1).

### Clinical conditions:

Most patients had genetically undefined conditions (n=17) and were divided into categories of clinically undefined (n=11), autoimmune cytopenias (n=6), or monogenic disorders of immune dysregulation (n=3). Eight of the 20 patients were determined to have active disease via chart review by the study’s immunologists, while the remaining 12 were quiescent.

### General Health Measure:

For the first question in the survey, patients were asked to rate their general health. No patients felt that their health was excellent, and two patients were unsure or skipped the question (Table 2). Patients otherwise ranged between very good to poor, with most patients rating their overall health as fair. There was no significant difference between the general health ratings of females versus males (*p*=0.875), and this response did not vary based on age group (*p*=0.565).

A comparison between the patient’s reported general health and cTfh percentage was also assessed (Table 2). The lowest median cTfh percentage corresponded to the “poor” category, and the highest was associated with a designation of “fair,” however there was no significant difference in cTfh percentage between each designation (*p*>0.999).

**Figure 1:**
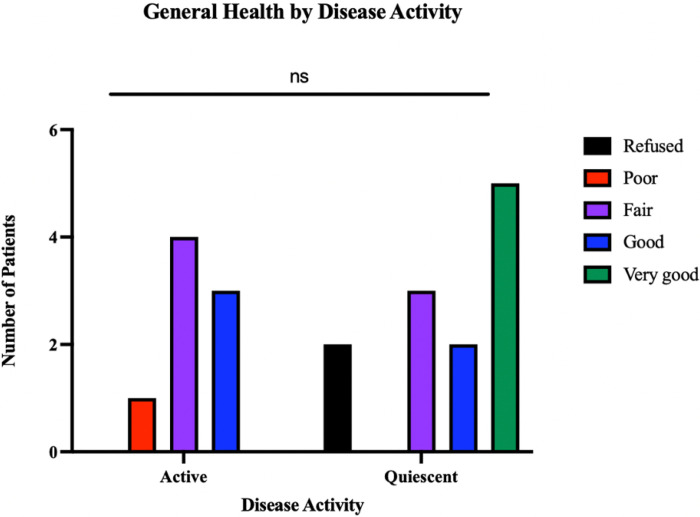
Patient-reported general health by disease activity. Patient-reported perceptions of their general health over the previous 30 days are shown, with the total number of patients divided by disease activity. Multiple comparisons Kruskal-Wallis test did not reveal any significant differences between the active versus quiescent disease responses in this category (*p*>0.05 for all comparisons).

There was no significant difference between the general health ratings based on disease activity (*p*=0.282), however 58.3% of those with quiescent disease reported good or very good overall health, whereas only 37.5% of those with active disease gave the same rating (Figure 1). When separated by disease category, there was no significant difference in reported general health (*p*=0.122).

### Unhealthy Days Measure:

The patient reported number of physically unhealthy days, mentally unhealthy days, and total unhealthy days in the preceding month are shown in Table 3. Patients who skipped questions or selected “unsure” or “refused” were left blank in the table. There was no significant difference in cTfh percentage between the three unhealthy day measurements (Table 3).

**Figure 2:**
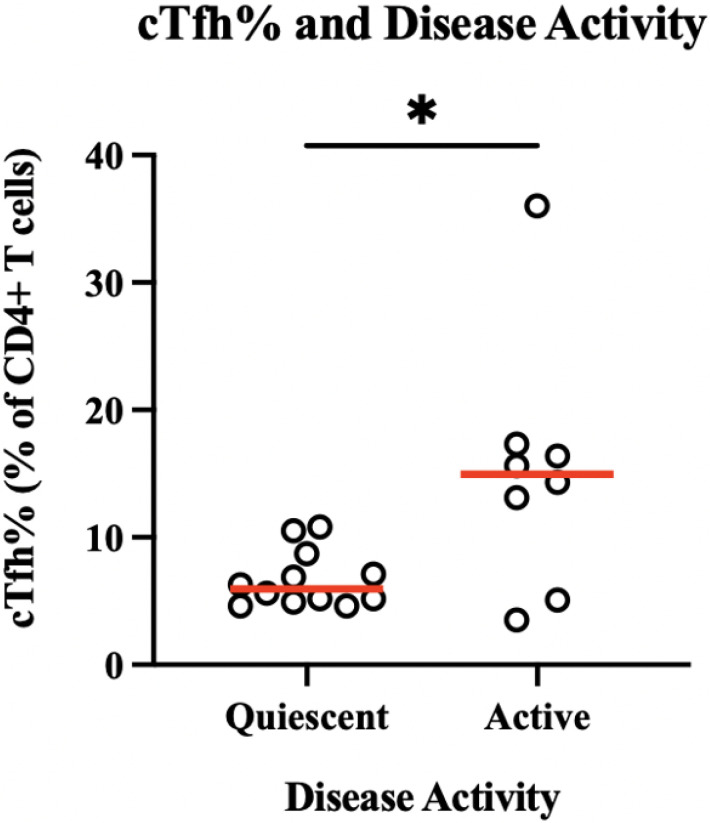
Circulating T follicular helper cell percentages by disease activity. T follicular helper cell levels are reported as percentage of CD4+ T cells and divided by a binary measurement of active versus quiescent disease. The median cTfh% is shown in red on the graph, and those with active disease had a significantly higher level by Mann-Whitney test, 14.95% versus 5.95%, respectively. *p=0.04

The cTfh percentages were compared between those with active versus quiescent disease, and those with active disease had significantly higher levels with a median of 14.95% of CD4+ T cells (Figure 2, *p*<0.05).

Patients with active disease were found to have a higher median number of physically unhealthy, mentally unhealthy, and total unhealthy days as compared to those with quiescent disease, however this difference was not significant (Table 4).

**Figure 3:**
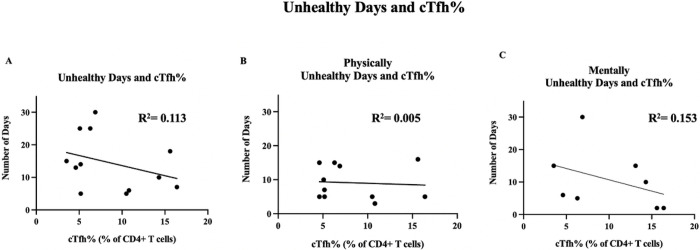
Correlation between circulating T follicular helper cell percentages and patient-reported unhealthy days. T follicular helper cell levels are reported as percentage of CD4+ T cells, as compared to the total number of unhealthy days out of the previous 30 days, *p*=0.689 (A); number of physically unhealthy days out of the previous 30 days, *p*=0.447 (B); and number of mentally unhealthy days out of the previous 30 days, *p*=0.188 (C). A simple linear regression was performed for each comparison, with R^2^ values shown on the graph. P values were not significant for any measurement, as calculated by Spearman correlation (*p*>0.05).

To determine if there existed a trend between cTfh percentage and number of total unhealthy days, physically unhealthy days, and mentally unhealthy days, simple linear regression with Spearman rank correlations were performed, as is shown in Figure 3 (A, B, and C, respectively). There was no obvious trend to the data (*p*>0.05), with R^2^ values shown in the figure for each respective measurement.

## Discussion

In our cohort and in others^13–16,18–21,25^, elevated cTfh percentages were shown to correlate with active immune dysregulation (*p*<0.05). However, this marker is not elevated in all patients with active disease, particularly in those with diagnoses such as ITP or some patients with common variable immune deficiency^22,26–29^. Multi-system organ damage can occur in many inborn errors of immunity and is a vital reason for achieving appropriate treatment^30^, but improving patient quality of life should also be something that all providers strive to achieve. Within our cohort, patients with active disease had a higher number of physically and mentally unhealthy days within a one month period, although this difference was not significant, and there was not a correlation between level of cTfh percentage and number of unhealthy days. A larger number of surveyed patients may change the significance of these findings, though further investigation is needed.

We chose to focus on the Healthy Days Core Module questions as this survey tool has been used for decades by multiple organizations for quality of life measurements, including in patients with chronic, waxing and waning health conditions^24^ (**Supplemental**). Furthermore, these first four questions focus on “the last 30 days” of the patient experience. As we aimed to assess the ability of a quality of life survey to reflect the level of disease activity, questions from the Activity Limitations Module were felt to be less relevant given the lack of a timeline. The questions within the Healthy Days Symptoms Module are specific to the one month timeline, however they address specific components of the patient’s mental and physical health and thus were not felt to add additional high yield information for our specific study question.

Regarding the Healthy Days Core questions in particular, only four out of twenty patients had a numeric response to question number 4, which asks how many days out of the previous 30 that poor physical or mental health prevented the patient from doing his or her usual activities (**Supplemental**). Of the four who did respond, the answer to this question was equivalent to either the total unhealthy days, physically unhealthy days, or mentally unhealthy days, respectively. It was therefore decided to exclude this question from statistical analysis with the significant amount of missing data, as well as the similarity to the answers to questions 2 and 3. It is possible that further investigation into this question would provide better insight into the patient experience; however, additional studies are needed to make this determination.

Of the total patients who completed the survey, 30% did not provide information regarding the number of unhealthy days in the preceding month. In the youngest age group, the mental health questions were likely not applicable to many, and therefore were skipped by parents. In older children and adolescents, it is known that many factors contribute to a child not revealing mental health concerns^31^. As the goal of this study was to evaluate the ability of a quality of life survey to identify active disease in patients with immune dysregulation, healthy controls were not included. However, with the current pediatric mental health crisis^32^, these specific questions could be affected. It could therefore be useful to include a health control cohort in future evaluations.

A major limitation of the study is a lack of adequate representation from historically marginalized groups. Given the uneven numbers, we were unable to perform accurate statistical analysis to determine if this was a contributing factor to the HRQOL results. It has been shown that race and ethnicity are not known to affect cTfh percentage alone^33^. However, social determinants of health are known to affect patient quality of life^34^, and this is an important area for further investigation within this patient population.

One reason for the lack of correlation could also be the choice of a survey tool. As discussed previously, few studies have sought to validate quality of life surveys in patients with IEI^6,35^, and these have focused on humoral immune deficiencies, primarily in adult patients. This survey was chosen for its broad utilization in a range of diagnoses, in addition to the simplicity and short length. However, immune dysregulation can be quite phenotypically variable, with a range of physical and mental challenges that are unlikely to be adequately represented in a simple survey. It has also been shown that in patients with fluctuating levels of disease control, it may be difficult for them to retrospectively assess their experiences over a prolonged period of time, even if this only a one month period^36^. This suggests that a different quality of life measurement tool may be needed in this patient population.

## Conclusions

Circulating T follicular helper cell percentage is a helpful way to measure disease activity in patients with immune dysregulation, however it does not reflect the patient’s health related quality of life. This is an important finding as other diagnostic modalities may be similarly discordant with the patient experience, despite an apparent understanding of the level of disease control by the patient’s treating physician. Although our sample size was small, the CDC HRQOL-14 does not appear to reflect disease activity in patients with immune dysregulation, which encompasses a complicated and diverse range of diagnoses. The development of HRQOL surveys under the guidance of patients themselves could be the next step for a more accurate and applicable tool, and this could subsequently be validated within the IEI population. Investigating a more diverse cohort, in addition to the inclusion of healthy controls, could provide a more thorough and accurate evaluation. Investigating HRQOL is a subject that is significantly lacking for patients with IEI, and we have the potential to improve patient outcomes with better investigation of this important aspect of the patient experience.

## Figures and Tables

**Table 1:** Patient Demographics

	Number of patients (n=20)
**Female sex**	12 (60%)
**Race and ethnicity**	
White, non-Hispanic	17 (85%)
Hispanic/Latino	2 (10%)
Black/African American, non-Hispanic	1 (5%)
**Age Groups, in years**	
1 – 6	5 (25%)
6 – 12	4 (20%)
12 – 16	4 (20%)
>16	7 (35%)

**Table 2:** General Health

	Very Good	Good	Fair	Poor	Unsure/Refused
**Patient Sex**					
Female (n=12)	3 (25%)	3 (25%)	4 (33.3%)	0	2 (16.7%)
Male (n=8)	2 (25%)	2 (25%)	3 (37.5%)	1 (12.5%)	0
**Patient Age Group, in years**					
1–6 (n=5)	1 (20%)	1 (20%)	3 (60%)	0	0
6–12 (n=4)	0	2 (50%)	1 (25%)	1 (25%)	0
12–16 (n=4)	2 (50%)	1 (25%)	1 (25%)	0	0
>16 (n=7)	2 (28.6%)	1 (14.3%)	2 (28.6%)	0	2 (28.6%)
**Patient Disease Activity**					
Active (n=8)	0	3 (37.5%)	4 (50%)	1 (12.5%)	0
Quiescent (n=12)	5 (41.7%)	2 (16.7%)	3 (25%)	0	2 (16.7%)
**Disease Category**					
Clinically undefined (n=11)	2 (18.2%)	2 (18.2%)	4 (36.4%)	1 (9.1%)	2 (18.2%)
Autoimmune cytopenias (n=6)	1 (16.7%)	2 (33.3%)	3 (50%)	0	0
Monogenic disorders (n=3)	2 (66.7%)	1 (33.3%)	0	0	0
**Median cTfh%**	8.73	5.18	13.1	5.08	5.75

*p*=ns for all categories

**Table 3:** Unhealthy Days Measure

	Disease Activity	cTfh% (% of CD4+ T cells)	Total Unhealthy Days (max 30)	Physically Unhealthy Days	Mentally Unhealthy Days
**Clinically undefined (n=11)**					
Lymphoprolifération, autoimmune cytopenias	Active	15.6%	18	16	2
Combined immune deficiency, autoimmune cytopenias	Active	14.3%	10	0	10
Autoimmune hepatitis, autoimmune cytopenias	Quiescent	10.8%	3	3	0
Pulmonary autoimmune granulomatous disease	Quiescent	10.5%	5	5	0
Humoral immune deficiency, autoimmune cytopenias, lymphoproliferation, GLILD	Quiescent	6.88%	30	14	30
Pulmonary nodules, autoimmune hepatitis, autoimmune neurologic disease	Quiescent	5.61%	NA		
Humoral immune deficiency, ILD	Quiescent	5.18%	5	5	0
Humoral immune deficiency, lymphoproliferation	Active	5.08%	10	10	0
Combined immune deficiency, autoimmune neurologic disease	Quiescent	4.92%	NA		
Combined immune deficiency, IBD	Quiescent	4.61 %	15	15	0
Humoral immune deficiency, ILD	Quiescent	4.61 %	11	5	6
**Autoimmune cytopenias (n=6)**					
Evans Syndrome	Active	36%	NA		
Evans Syndrome	Active	17.3%	NA		
Evans Syndrome	Active	13.1%	15	0	15
Evans Syndrome	Quiescent	8.73%	NA		
Autoimmune neutropenia	Quiescent	5.17%	7	7	0
ITP	Active	3.53%	15	0	15
**Monogenic disorders (n=3)**					
Activated PI3K Delta Syndrome	Active	16.4%	7	5	2
CTLA-4 haploinsufficiency	Quiescent	7.12%	NA		
*AIRE* gene variant	Quiescent	6.29%	20	15	5

GLILD: granulomatous lymphocytic interstitial lung disease; IBD: inflammatory bowel disease; ILD: interstitial lung disease; ITP: immune thrombocytopenia

**Table 4:** Disease Activity with cTfh Percentage and Unhealthy Days

	Median cTfh%	Median Total Unhealthy Days	Median Physically Unhealthy Days	Median Mentally Unhealthy Days
**Active disease (n=8)**	14.95 (3.53–36)	12.5 (7–18)	7.5 (0–16)	10 (2–15)
**Quiescent disease (n=12)**	5.95 (4.61–10.8)	9.0 (3–30)	6.0 (3–15)	6.0 (5–30)
**P value**	*p*=0.04	*p*=0.591	*p*=0.970	*p*=0.714

## Data Availability

The datasets used and/or analyzed during the current study are available from the corresponding author on reasonable request.

## Abbreviations

CDC: Centers for Disease Control
cTfh: circulating T follicular helper
HRQOL: health-related quality of life
IEI: inborn errors of immunity
ITP: immune thrombocytopenia
